# Factors Associated with Clinical Research Recruitment in a Pediatric Academic Medical Center—A Web-Based Survey

**DOI:** 10.1371/journal.pone.0140768

**Published:** 2015-10-16

**Authors:** Erica Rose Denhoff, Carly E. Milliren, Sarah D. de Ferranti, Sarah K. Steltz, Stavroula K. Osganian

**Affiliations:** 1 The Clinical Research Center, Boston Children’s Hospital, Boston, MA, United States of America; 2 Department of Cardiology, Boston Children’s Hospital, Boston, MA, United States of America; 3 New Balance Obesity Prevention Center, Boston Children’s Hospital, Boston, MA, United States of America; 4 Division of General Pediatrics, Department of Medicine, Boston Children’s Hospital, Boston, MA, United States of America; 5 Division of Endocrinology, Department of Medicine, Boston Children’s Hospital, Boston, MA, United States of America; University Hospital Lausanne, SWITZERLAND

## Abstract

**Background:**

One of the most difficult aspects of conducting clinical research is the ability to successfully recruit participants. Pediatric clinical research presents unique recruitment challenges that relate to the need for parental consent on behalf of a minor, child assent, and school attendance. Yet, this has been less well studied. We conducted a survey of investigators performing human subjects research in a single large academic pediatric hospital to better understand characteristics of studies with successful recruitment.

**Methods:**

We conducted a web-based survey from September 2011 to December 2011 of all principal investigators with an Institutional Review Board approved human subjects protocol at Boston Children’s Hospital, a pediatric Academic Medical Center. The survey captured various characteristics of the protocols including study design, staffing, resources, and investigator experience and training as well as respondents’ perceived barriers and facilitators to recruitment. We used chi square tests and Mantel-Haenszel test for linear trend to examine the relationship between selected predictor variables and the binary outcome of successful vs. unsuccessful recruitment and multivariable logistic regression analyses to examine the simultaneous influence of potential predictors on each outcome.

**Results:**

Among the 349 eligible investigators, 52% responded to the survey, and 181 with valid data were included in the analyses. Two-thirds of the 87 protocols closed to enrollment reached 80% or more of their target enrollment, whereas, only one-third of the 94 protocols actively recruiting were meeting 80% of their target. Recruitment method appeared to be the only significant and independent factor associated with achieving 80% or more of target enrollment in closed to enrollment protocols. Closed to enrollment protocols that used recruitment in person were 4.55 times (95% CI 1.30 to 15.93; p = 0.02) more likely to achieve 80% or more of their target enrollment when compared to those that used other recruitment methods. Other potentially modifiable factors such as number of study visits, study duration and investigator experience were suggestive of being meaningfully related to recruitment.

**Conclusion:**

Recruiting in person may promote reaching an acceptable target enrollment in pediatric as well as adult clinical research. Future research is needed on larger and more diverse samples to gain a better understanding of how the characteristics and qualifications of the individuals who conduct recruitment influence participant enrollment as well as how best to approach patient and families for their participation.

## Background

One of the most challenging aspects of conducting clinical research is the ability to successfully recruit participants. The inability to recruit the target sample size has been estimated to occur in approximately 80% of clinical trials [1]. The impact of low recruitment to a study can be serious, leading to early termination with insufficient sample size and subsequent losses in statistical power and limited generalizability [2,3]. In addition, slower than anticipated recruitment may increase the duration of the study, delaying the reporting of results and causing unanticipated stress on the budget and resources [2].

Clinical trials are particularly susceptible to low recruitment rates because they often require greater participant burden or are higher risk [2]. A number of factors such as patient concerns about safety, unwillingness to be randomized, investigator inexperience, inadequate staff efforts or logistical problems with protocol implementation have been associated with inadequate participant accrual in adult investigations [4,5]. Pediatric clinical research presents recruitment challenges above and beyond those affecting clinical research in adult populations [6]. Parents or guardians as well as children are involved in the decision-making about participation. Children may also have less availability because they are avoiding school absences, and working parents may have trouble taking time off from work. Increasing clinical trial participation rates is particularly urgent for pediatrics because children have historically been an understudied group, with about 75% of drugs lacking appropriate pediatric pharmacokinetic and safety data [6]. As a result, physicians are often forced to use off-label prescriptions for children based on adult data [6]. Lack of evidence-based data in youth or untimely reporting of research results may delay the availability of potentially effective treatments [5].

A substantial body of literature has examined characteristics of low enrolling studies and barriers and facilitators to participation in adult clinical research, but pediatric clinical research is less well studied [7,8]. Understanding the challenges of recruiting participants into pediatric clinical research is essential to helping investigators and their teams more effectively design and implement studies with children and adolescents. We conducted a survey of investigators performing human subjects research in a single large academic children’s hospital to better understand characteristics of studies with successful recruitment.

## Methods

### Design

The Institutional Review Board (IRB) at Boston Children's Hospital approved this research under expedited review on January 26, 2011; IRB approval protocol number X11-01-0015. We conducted a cross-sectional, web-based survey (S2 File) of all researchers listed as the ‘principal investigator’ on an IRB-approved human subjects protocol registered with the Committee on Clinical Investigation (CCI) at Boston Children’s Hospital (BCH). A list of principal investigators and active protocols were generated from the CCI database at two time points in July, 2010 and June, 2011 with submission dates beginning in 1987. If an investigator had more than one protocol registered with the CCI, one protocol was randomly selected for inclusion in the survey. The principal investigator was then emailed an invitation to participate with a hyperlink to complete the survey or refuse participation. Two reminder emails were sent to non-respondents at two weeks and three weeks after the initial email invitation. A hard copy of the survey was sent to the remaining non-respondents 2 weeks after the final email reminder to provide the option to complete the survey on hard copy and return it by interoffice mail. Participation was completely voluntary and informed consent was not required. A $20 gift card was provided as a token of appreciation to all respondents.

We developed a 31 item structured questionnaire that included questions adapted from two existing instruments on the topic of participant recruitment [9,10].(See Appendix) The questionnaire included items on the following potential predictors of recruitment: type of study design, type of intervention, frequency and duration of a study visit, duration of follow-up, severity of disease and, IRB designated risk/benefit determination, location of study visit, recruitment methods (including in person recruitment where research staff directly approach potentially eligible families and children, telephone calls, mailings, email contact, or use of media for advertising). participant demographics, incentive or token of appreciation provided, project staffing and budget, and investigator and study staff characteristics (academic rank, education, years of experience, and percent PI or coordinator work effort devoted to the project based on a full time work equivalent of 100%). Investigators’ attitudes and beliefs about barriers and facilitators to recruitment specific to the protocol being surveyed were assessed on a 4-point likert scale a strongly agree, agree, disagree and strongly disagree. The investigators were also asked to report the target sample size, date recruitment began, date recruitment ended or was projected to end, as well as the number of participants enrolled in the study as of the date the survey administration (for studies actively recruiting) or as of the date the study was closed to enrollment (for studies that had completed recruitment). Ten additional questions were included that captured characteristics of the investigator such as age, gender, education, academic rank, and years of training and experience in clinical research. The questionnaire was first piloted with a convenience sample of 10 investigators for face validity. Minor and non-substantive changes were made to improve clarity. The average time to complete the survey was 20 minutes, and the web-based survey was administered from September 2011 to December 2011 using SPSS (PASW Data Collection version 5.6).

Three hundred and eighty one investigators were emailed an invitation to participate in the web-based survey. Among these, 15 were no longer working at BCH and thus ineligible to participate. One investigator declined to participate, and 166 did not respond. Among those who responded, 17 reported that their study did not require recruitment of participants and were not eligible to participate. Among the remaining 349 eligible investigators, 52% responded to the survey (n = 182). A survey was considered complete for analysis if the respondent progressed through the first 28 questions that addressed enrollment and study characteristics, but may have stopped before completing the final sections on attitudes, beliefs, and respondent characteristics. The majority (n = 177) of the respondents progressed through the entire survey. Only 22 respondents submitted the survey on hard copy.

About half (56%) of the respondents were female. Two thirds (66%) were physician investigators, and 21.5% were some other type of clinician investigator such as nurses, dentists, or psychologists. Most (81%) had faculty appointments at Harvard Medical School. The number of years of having been a principal investigator on a human subjects’ protocol varied widely and ranged from 0 to 38 years (Mean 8.4; SD 8.5 years).

## Statistical Analysis

The distribution of respondent and study characteristics were described by means, medians and frequencies. In bivariate analyses, we used a Chi Square test of association or Mantel-Haenszel test for linear trend (for ordinal variables) to examine the relationship between categorical predictor variables and the binary outcome of successful vs. unsuccessful recruitment. The primary outcome of successful recruitment was defined a priori as enrollment of 80% or more of the target sample size at completion of enrollment, or for studies actively recruiting, 80% or more of the expected enrollment at the time of the survey administration. For actively recruiting studies, we calculated the expected enrollment at the time of the survey by multiplying together the projected average recruitment rate and duration of recruitment as of the time of survey. For example, a study with a 10 month recruitment timeline that had to enroll 100 subjects during this time period would have a projected average recruitment rate of 10 subjects per month. If the survey was completed at month 5 of their recruitment timeline then the expected enrollment at month 5 would be 50 subjects (10 subjects/month x 5 months). If the actual enrollment was only 25 subjects at month 5, then this study would have met only 50% (25/50 x 100) of their expected enrollment. Given that some studies with low enrollment can still provide useful descriptive data on feasibility, effect size estimates, and safety data, we examined enrollment of 50% or more of the target sample size as a secondary and exploratory outcome of interest. To examine the simultaneous influence of potential predictors on each outcome, we conducted a multivariable logistic regression analysis that included all variables with a p-value <0.1 in bivariate analyses (S1 File). These models were also adjusted for type of study design (observational vs. interventional). All independent variables were entered into the equation in one step, using the forced entry method.

The responses to statements on barriers and facilitators were combined into two categories such that agreement was defined as “strongly agree or agree” and disagreement was defined as “strongly disagree or disagree”. All analyses were conducted separately for protocols that were closed to enrollment vs. protocols actively recruiting. We excluded from the analyses one respondent whose percent target enrollment was calculated as 600% and thus considered an implausible value. Therefore, the analyses were conducted with 181 completed survey forms. Data were analyzed in SAS (version 9.2). A two-sided p-value < 0.05 was considered statistically significant.

## Results

### Characteristics of Protocols

**Fig 1:**
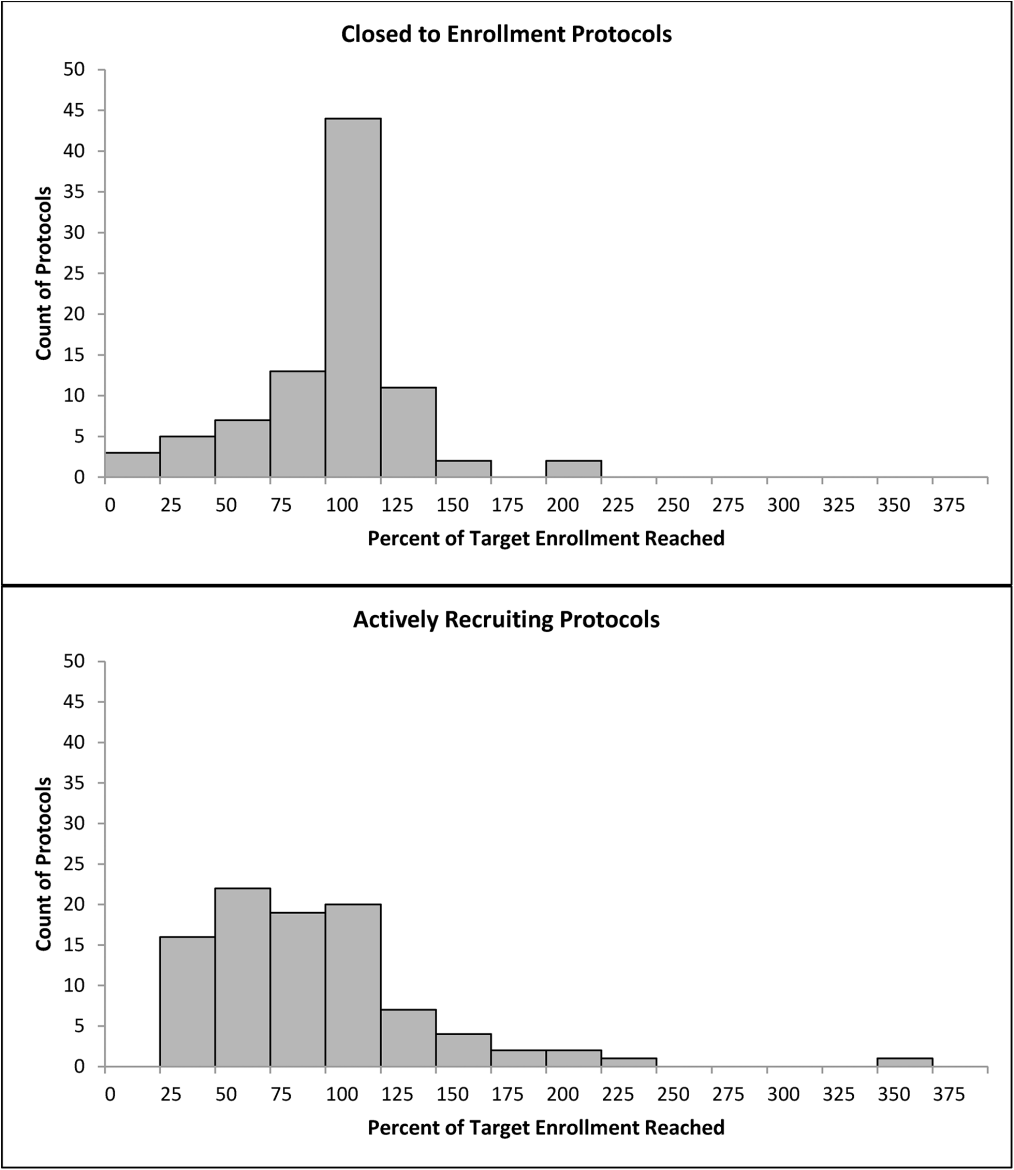
Frequency distribution of the percent of target enrollment for actively recruiting (n = 94) and closed to enrollment (n = 87) protocols.

**Fig 2:**
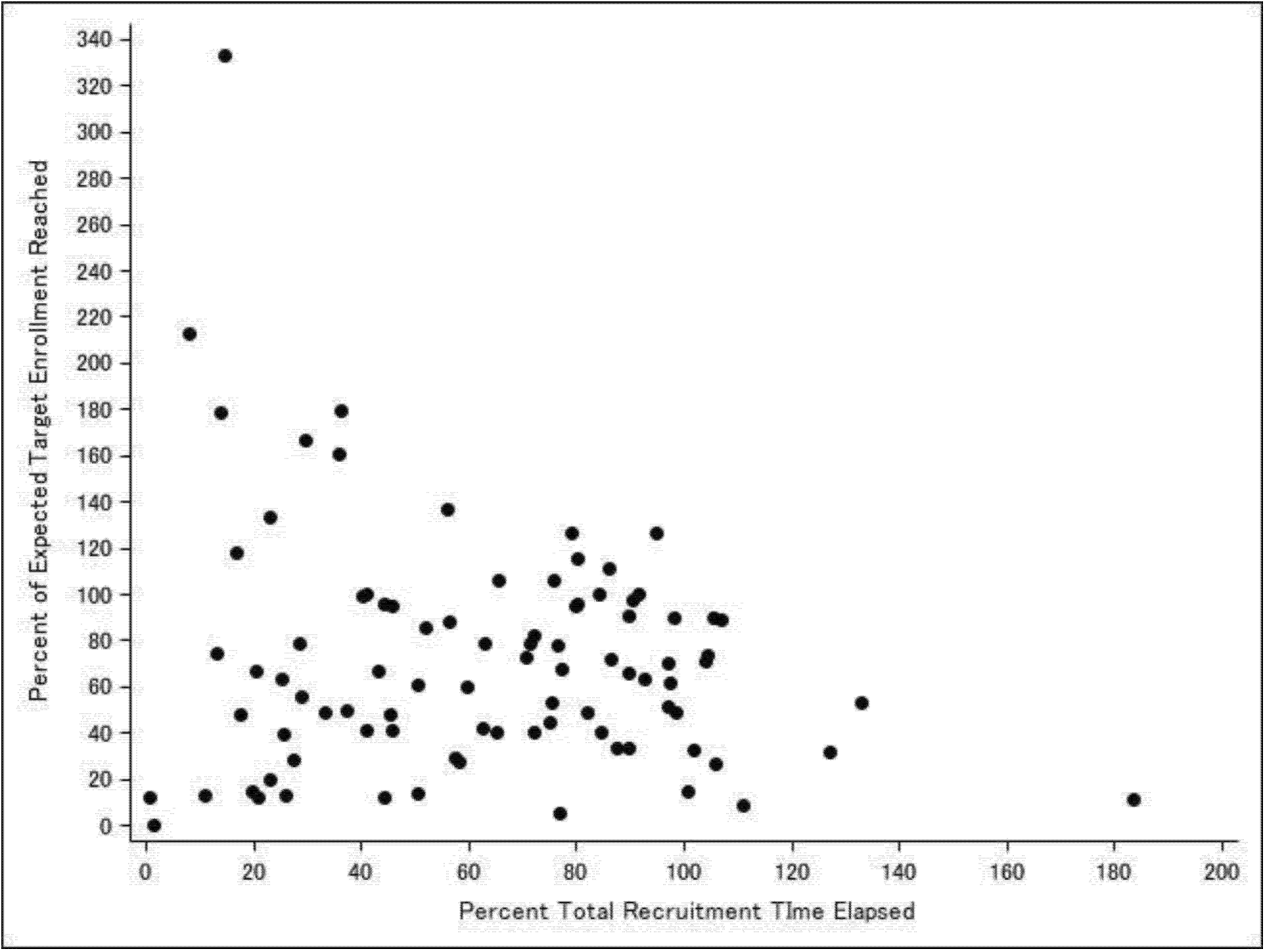
Scatterplot of percent of expected target enrollment reached by percent of total recruitment time elapsed for actively recruiting protocols.

Eighty-seven investigators (48.1%) reported that their protocols were closed to enrollment; while 94 investigators (51.9%) reported that their protocols were actively recruiting participants at the time the survey was completed (Table 1). The majority (67.4%) of the 181 protocols were observational studies. Among the 59 clinical trials, 42.4% were drug trials; 15.3% were device trials; and 32.2% were behavioral interventions. The study visits took place in multiple settings but primarily in the clinical settings including hospital ambulatory clinics (45.7%), off site or satellite ambulatory clinics (24.6%), inpatient units (35.9%), or Clinical and Translational Study Unit (16.9%). Nearly half of all protocols (46.1%) reported that they had experienced delays in their study recruitment timeline ranging from 1 to 36 months (median delay 6 months; interquartile range 4 to 12 months). Overall, approximately half (49.7%) of all protocols achieved 80% or more of their target enrollment. The distribution of percent of target enrollment for closed to enrollment and actively recruiting protocols is shown in Fig 1. For those protocols that were closed to enrollment, two-thirds (65.5%) reached 80% or more of their target enrollment; whereas substantially fewer (35.1%) protocols that were actively recruiting had reached 80% or more of their expected target enrollment based on their anticipated recruitment timeline. Fig 2 shows that actively recruiting protocols were widely dispersed at times along their recruitment timeline. A relatively greater number of protocols were falling short of 80% of their expected target enrollment at times beyond the midpoint of their timeline when compared to times earlier than the midpoint; 38 (40.4%) vs. 23 (24.5%) protocols, respectively.

**Table 1:** Characteristics of Included Protocols (n = 181).

Study Characteristic	All Protocols	Closed to Enrollment	Actively Recruiting
		Protocols	Protocols
	N	n	n
**Total Number**	181	87	94
**Achieved 80% or more of enrollment goal**	90 (49.7%)	57 (65.5%)	33 (35.1%)
**Experienced Delays in Recruitment Timeline**	82 (46.1%)	33 (37.9%)	49 (53.8%)
**Study Design**			
Clinical Trial	59 (32.6%)	30 (34.5%)	29 (30.9%)
Drug Trial	25 (42.4%)	17 (56.7%)	8 (27.6%)
Device Trial	9 (15.3%)	0	9 (31%)
Behavioral Intervention	19 (32.2%)	10 (33.3%)	9 (31.0%)
Other	6 (3.3)	3 (10.0%)	3 (10.3%)
Observational	122 (67.4%)	57 (65.5%)	65 (69.1%)
**Location of Study Visits** *			
Hospital Ambulatory Clinics	80 (45.7%)	31 (37.3%)	49 (53.3%)
Off Site or Satellite Ambulatory Clinics	42 (24.6%)	14 (17.2%)	28 (31.2%)
Hospital Inpatient Units	65 (35.9%)	23 (26.4%)	42 (44.7%)
Hospital-based Clinical and Translational Study Unit	29 (16.9%)	14 (17.1%)	15 (16.7%)
Home or Community Setting	15 (8.8%)	8 (9.9%)	7 (7.8%)
Survey (email, mail, web)	22 (12.7%)	11 (13.4%)	11 (12.1%)
Other (not specified)	17 (9.9%)	8 (9.8%)	9 (10.0%)
Median Delay in Recruitment Timeline(min, 25^th^%ile, 75^th^%ile, max)	6.00 months (1, 4, 12, 36)	12.00 months (2, 6, 12, 24)	6.00 months (1, 3, 12, 36)

*Categories are not mutually exclusive as some study visits take place in more than one location

### Findings for Protocols Closed to Enrollment

Table 2 shows the association of various study characteristics with meeting enrollment targets (≥ 80%) for those protocols that were closed to enrollment. Participant age, study design, visit duration, risk of death or disability associated with the disease or condition under evaluation, and protocol risk showed no consistent or statistically significant association with reaching 80% or more of the target enrollment. The only factors that tended to significantly favor 80% or more of target enrollment were research studies that required one study visit (65% vs. 43%, respectively; p = 0.05) or recruited in person (86% vs.70% respectively; p = 0.07). Various study resources and investigator characteristics, including incentive value, coordinator and principal investigator work effort, and training and experience showed no statistically significant trends or associations with reaching 80% or more of target enrollment (Table 3). However, among studies reaching 80% of target enrollment, a higher percentage of studies did not have funding when compared to those that did not reach the 80% of target (32% vs. 13%, respectively; p = 0.06).

**Table 2:** Association between study characteristics and reaching ≥ 80% of target enrollment among closed to enrollment protocols.

Study Characteristics	Below 80% of target	80% or above Target	p-value*
**Study Design**			0.21
Clinical Trial	13 (43%)	17 (30%)	
Observational	17 (57%)	40 (70%)	
**Randomized Trial** **			
Yes	8 (62%)	8 (47%)	0.43
No	5 (38%)	9 (53%)	
**IRB Designated Risk/ Benefit Determination**			0.75*
No More than Minimal Risk, Direct Benefit	10 (33%)	11 (19%)	
No More than Minimal Risk, No Direct Benefit	12 (40%)	36 (63%)	
Greater than Minimal Risk	8 (27%)	10 (18%)	
**Risk of Death/Disability of Disease**			0.34*
High	7 (23%)	12 (21%)	
Moderate	8 (27%)	22 (39%)	
Minimal to None	5 (17%)	13 (23%)	
No disease	10 (33%)	10 (18%)	
**Number of Study Visits**			0.05
One Visit	13 (43%)	37 (65%)	
More than one Visit	17 (57%)	20 (35%)	
**Visit Duration**			0.21*
0–1 hours	14 (47%)	21 (37%)	
>1 to 2 hours	7 (23%)	10 (18%)	
>2 hours	9 (30%)	23 (46%)	
**Study Duration**			0.19
30 days or less	18 (60%)	42 (74%)	
More than 30 days	12 (40%)	15 (26%)	
**Recruitment method**			0.07
In person	21 (70%)	49 (86%)	
Other	9 (30%)	8 (14%)	
**Minimum Age of Participants**			0.91*
0–1 years	10 (34%)	17 (30%)	
2–4 years	2 (7%)	9 (16%)	
5–7 years	5 (17%)	9 (16%)	
8–12 years	6 (21%)	9 (16%)	
13or more years	6 (21%)	12 (21%)	
**Maximum Age of Participants**			0.91*
0–12 years	5 (18%)	10 (20%)	
13–18 years	12 (43%)	18 (35%)	
19–25 years	3 (11%)	12 (24%)	
26 or more years	8 (29%)	(22%)	

*P-values calculated using Chi Square tests of associations or Mantel Haenszel test for linear trend where designated with an asterisk

**Subgroup analysis limited to clinical trials

**Table 3:** Association between resources / staffing and reaching ≥ 80% of target enrollment among closed to enrollment protocols.

Study Resources and Staff	Below 80%	At 80% or above	p—value*
	of target	target	
**Incentive Value**			0.24*
None	14 (47%)	30 (53%)	
>0 to $20	2 (7%)	8 (14%)	
>$20 to $50	6 (20%)	8 (14%)	
More than $50	8 (27%)	11 (19%)	
**Funding**			0.06
Funded	26 (87%)	39 (68%)	
Not Funded	4 (13%)	18 (32%)	
**Coordinator Work Effort**			0.39*
None	12 (40%)	17 (30%)	
>0 to 50%	11 (37%)	24 (42%)	
>50%	7 (23%)	16 (28%)	
**Coordinator Experience**			0.50*
No Experience	3 (17%	9 (23%)	
>0 to 5 years	13 (72%)	23 (58%)	
More than 5 years	2 (11%)	8 (20%)	
**Principal Investigator Work Effort**			0.84*
Less than 5%	7 (23%)	14 (25%)	
5% to <10%	6 (20%)	7 (12%)	
10% to <15%	8 (27%)	14 (25%)	
15% to <25%	2 (7%)	13 (23%)	
25% or more	7 (23%)	9 (16%)	
**Faculty Rank**			.0.43*
Instructor	7 (23%)	15 (27%)	
Assistant Professor	11 (37%)	19 (35%)	
Associate/Full Professor	5 (17%)	15 (27%)	
No Faculty Appointment	7 (23%)	6 (11%)	
**Clinical Research Training**			0.31
None	2 (7%)	10 (18%)	
Doctoral	12 (40%)	14 (25%)	
Masters	5 (17%)	13 (23%)	
Seminars	11 (37%)	20 (35%)	
**Years Conducting Human Subjects Research**			0.70*
0 to 4 years	6 (20%)	12 (21%)	
5 to 9 years	12 (40%)	17 (30%)	
10 or more years	12 (40%)	27 (48%)	
**Years served as Principal Investigator**			0.10*
0 to 1 year	7 (23%)	6 (11%)	
2 to 4 years	6 (20%)	13 (23%)	
5 to 9 years	10 (33%)	14 (25%)	
10 or more years	7 (23%)	23 (41%)	

*P-values calculated using Mantel Haenszel test for linear trend were designated with an asterisk

In multivariable logistic regression models that included funding status, recruitment method, and number of study visits adjusted for study design, only recruiting method remained strongly and significantly associated with reaching 80% of target enrollment. Protocols that used recruitment in person were 4.55 times (95% CI 1.30 to 15.93; p = 0.02) more likely to achieve 80% or more of the target enrollment when compared to those using other methods of recruitment. (See Appendix).

### Sensitivity Analysis

When we examined how these same factors were associated with reaching 50% of target enrollment, only recruitment method and the principal investigator’s years of experience conducting clinical research showed statistically significant associations (Data not shown). Reaching vs. not reaching 50% of the target enrollment had a greater percentage of studies that used recruitment in person (86% vs. 53%, respectively; p = 0.004) and principal investigators with 10 or more years of experience (51% vs. 20%, respectively; p = 0.04). In multivariable logistic regression models that included recruitment method and principal investigator years of clinical research experience adjusted for study design, only recruitment method remained strongly and significantly associated with reaching 50% of target enrollment. Protocols that used recruitment in person were 9.07 times (95% CI 2.09 to 39.35; p = 0.003) more likely to achieve 50% of target enrollment when compared to those using other methods of recruitment.(See Appendix)

### Findings for Protocols Actively Recruiting

Table 4 shows the association of various study characteristics with meeting enrollment targets (≥ 80%) for those protocols that were actively recruiting at the time of the survey. Study design, visit number or duration, recruitment method, participant age, risk of death or disability associated with the disease or condition under evaluation, and protocol risk showed no consistent or statistically significant association with reaching 80% or more of the target enrollment. Various study resources and investigator characteristics, including incentive value, coordinator and principal investigator work effort, and training and experience showed no statistically significant trends or associations with reaching 80% or more of target enrollment (Table 5). When we examined how these same factors were associated with reaching 50% of target enrollment, there were similarly no significant differences for any of the study or investigator characteristics in Tables 4 or 5 between those who reached 50% of the enrollment target and those who did not. (Data not shown).

**Table 4:** Association between study characteristics and reaching ≥ 80% of target enrollment among actively recruiting protocols.

Study Characteristics	Below 80% of target	80% or above target	p-value*
**Study Design**			0.93
Clinical Trial	19 (31%)	10 (30%)	
Observational	42 (69%)	23 (70%)	
**Randomized Trial** **			0.89
Yes	10 (53%)	5 (50%)	
No	9 (47%)	5 (50%)	
**IRB Designated Risk/Benefit Determination**			0.53*
No More than Minimal Risk, Direct Benefit	15 (25%)	11 (33%)	
No More than Minimal Risk, No Direct Benefit	41 (67%)	19 (58%)	
Greater than Minimal Risk	5 (8%)	3 (9%)	
**Risk of Death/Disability of Disease**			0.72*
High	13 (21%)	5 (15%)	
Moderate	19 (31%)	13 (39%)	
Minimal to None	15 (25%)	6 (18%)	
No disease	14 (23%)	9 (27%)	
**Number of Study Visits**			0.50
One Visit	43 (70%)	21 (64%)	
More than one Visit	18 (30%)	12 (36%)	
**Visit Duration**			0.24*
0–1 hours	22 (36%)	16 (48%)	
>1 to 2 hours	10 (16%)	5 (15%)	
>2 hours	29 (48%)	12 (36%)	
**Study Duration**			0.44
30 days or less	47 (77%)	23 (70%)	
More than 30 days	14 (23%)	10 (30%)	
**Recruitment Method**			0.48
In Person	51 (85%)	28 (90%)	
Other	9 (15%)	3 (10%)	
**Minimum Age Participants**			0.20*
0–1 years	34 (57%)	15 (48%)	
2–4 years	9 (15%)	2 (6%)	
5–7 years	5 (8%)	4 (13%)	
8–12 years	8 (13%)	7 (23%)	
13or more years	4 (7%)	3 (10%)	
**Maximum Age of Participants**			0.15*
0–12 years	12 (20%)	5 (16%)	
13–18 years	16 (27%)	15 (48%)	
19–25 years	8 (14%)	6 (19%)	
26 or more years	23 (39%)	5 (16%)	

*P-values calculated using Mantel Haenszel test for linear trend were designated with an asterisk

**Subgroup analysis limited to clinical trials

**Table 5:** Association between resources and staffing and reaching ≥ 80% of target enrollment among actively recruiting protocols.

Study Resources and Staff	Below 80%	At 80% or above	p-value*
	of target	target	
**Incentive Value**			0.67
None	33 (54%)	16 (48%)	
>0 to $20	12 (20%)	6 (18%)	
>$20 to $50	7 (11%)	7 (21%)	
More than $50	9 (15%)	4 (12%)	
**Funding**			0.82
Funded	42 (70%)	21 (68%)	
Not Funded	18 (30%)	10 (32%)	
**Coordinator Work Effort**			0.13
None	21 (35%)	6 (19%)	
>0 to 50%	25 (42%)	12 (39%)	
>50%	14 (23%)	13 (42%)	
**Coordinator Experience**			0.83*
No Experience	10 (26%)	6 (24%)	
>0 to 5 years	24 (62%)	17 (68%)	
More than 5 years	5 (13%)	2 (8%)	
**Principal Investigator Work Effort**			0.25*
Less than 5%	11 (18%)	5 (16%)	
5% to <10%	21 (35%)	9 (29%)	
10% to <15%	11 (18%)	3 (10%)	
15% to <25%	8 (13%)	7 (23%)	
25% or more	9 (15%)	7 (23%)	
**Faculty Rank**			0.99*
Instructor	16 (27%)	10 (32%)	
Assistant Professor	14 (24%)	7 (23%)	
Associate/Full Professor	17 (29%)	5 (16%)	
No Faculty Appointment	12 (20%)	9 (29%)	
**Clinical Research Training**			0.94
None	13 (21%)	6 (18%)	
Doctoral	11 (18%)	7 (21%)	
Masters	11 (18%)	7 (21%)	
Seminars	26 (43%)	13 (39%)	
**Years Conducting Human Subjects Research**			0.88*
0 to 4 years	16 (27%)	9 (29%)	
5 to 9 years	18 (30%)	7 (23%)	
10 or more years	26 (43%)	15 (48%)	
**Years served as Principal Investigator**			0.33*
0 to 1 year	15 (25%)	10 (32%)	
2 to 4 years	13 (22%)	9 (29%)	
5 to 9 years	10 (17%)	2 (6%)	
10 or more years	22 (37%)	10 (32%)	

*P-values calculated using Mantel Haenszel test for linear trend were designated with an asterisk

### Investigators Attitudes about Barriers and Facilitators to Recruitment Success

**Fig 3:**
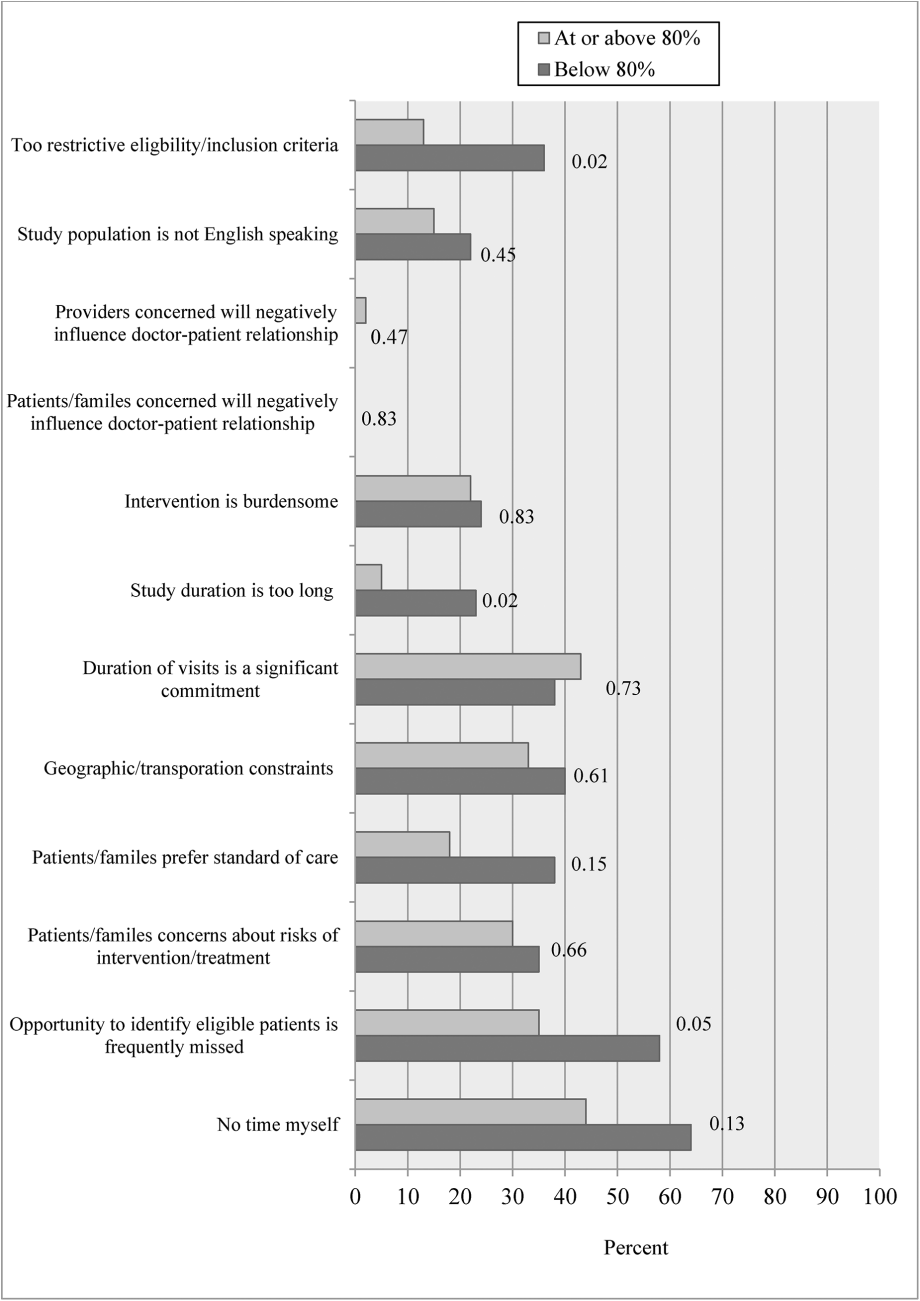
Percent of investigators agreeing with each barrier according to reaching target enrollment among closed to enrollment protocols.

**Fig 4:**
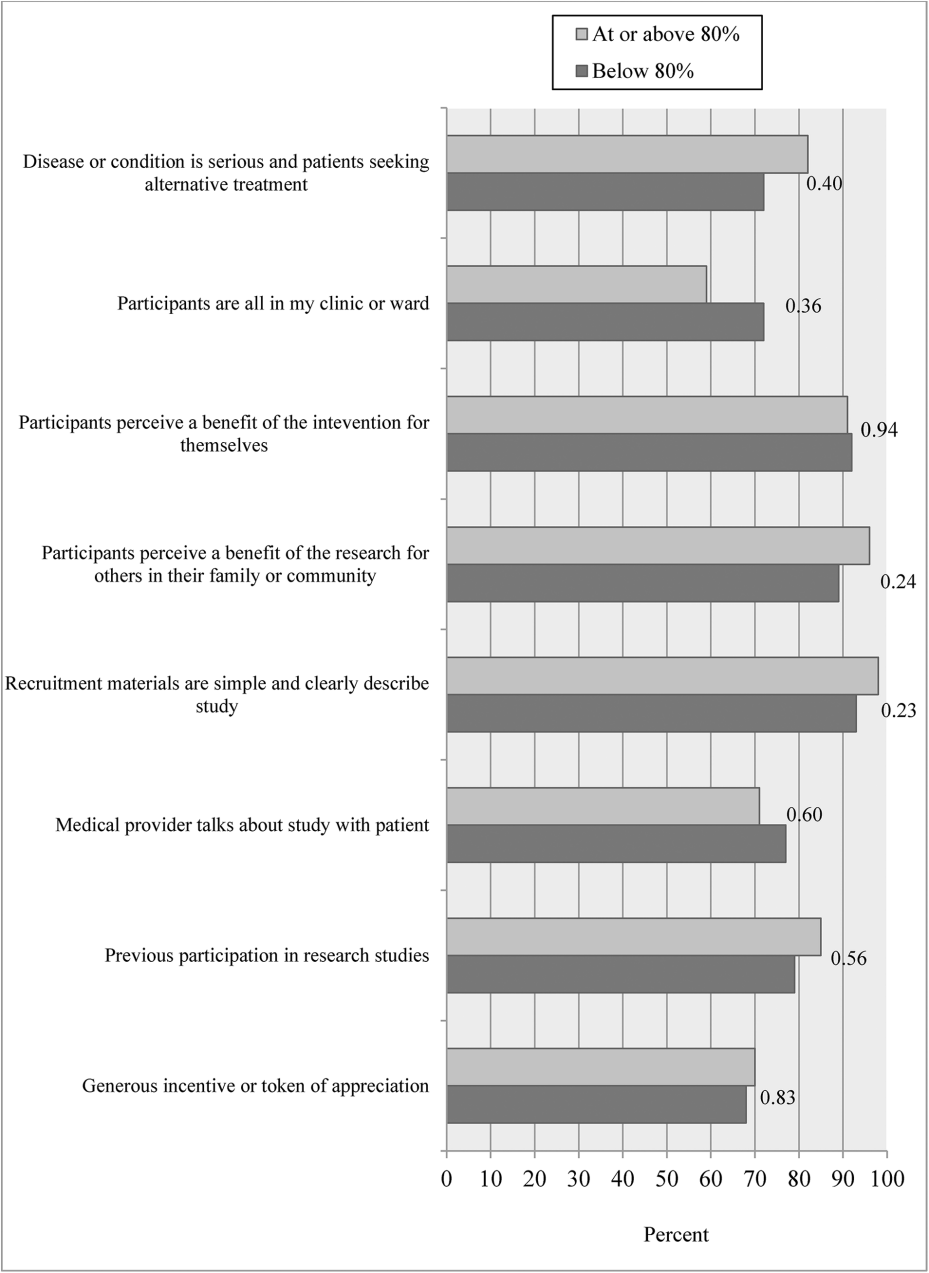
Percent of investigators agreeing with each facilitator according to reaching target enrollment among closed to enrollment protocols.

We asked investigators to report their perceptions about what made recruitment difficult in their specific study for protocols. Among closed to enrollment studies (Fig 3), only two perceived barriers, “long study duration” and “too restrictive eligibility criteria” showed statistically significant differences between studies that reached vs. did not reach their 80% target enrollment. A high proportion of investigators agreed with the majority of facilitators to recruitment shown in Fig 4, and none of the facilitators differed significantly between those who reached vs. did not reach their 80% target.

**Fig 5:**
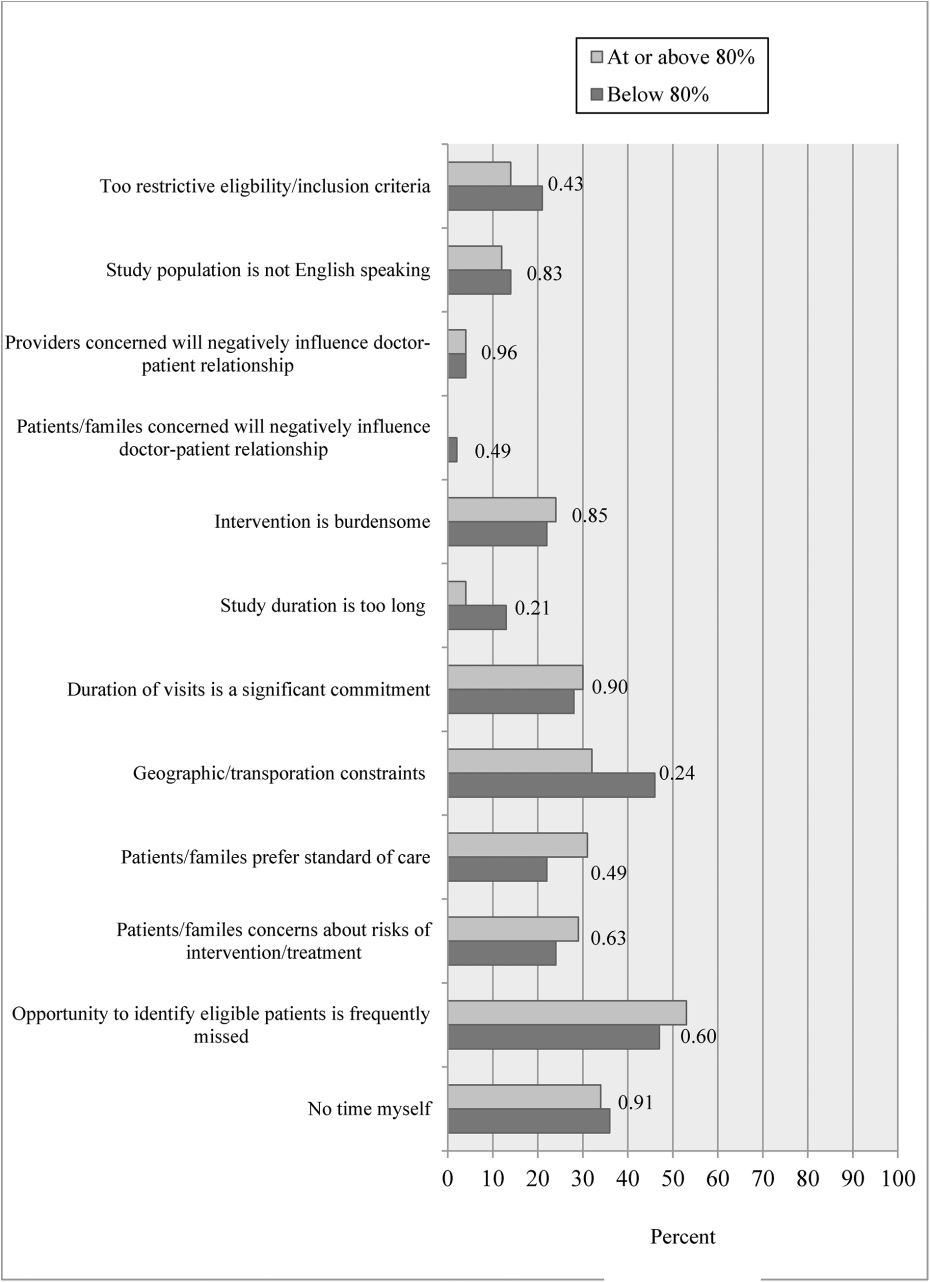
Percent of investigators agreeing with each barrier according to reaching target enrollment among actively recruiting protocols.

**Fig 6:**
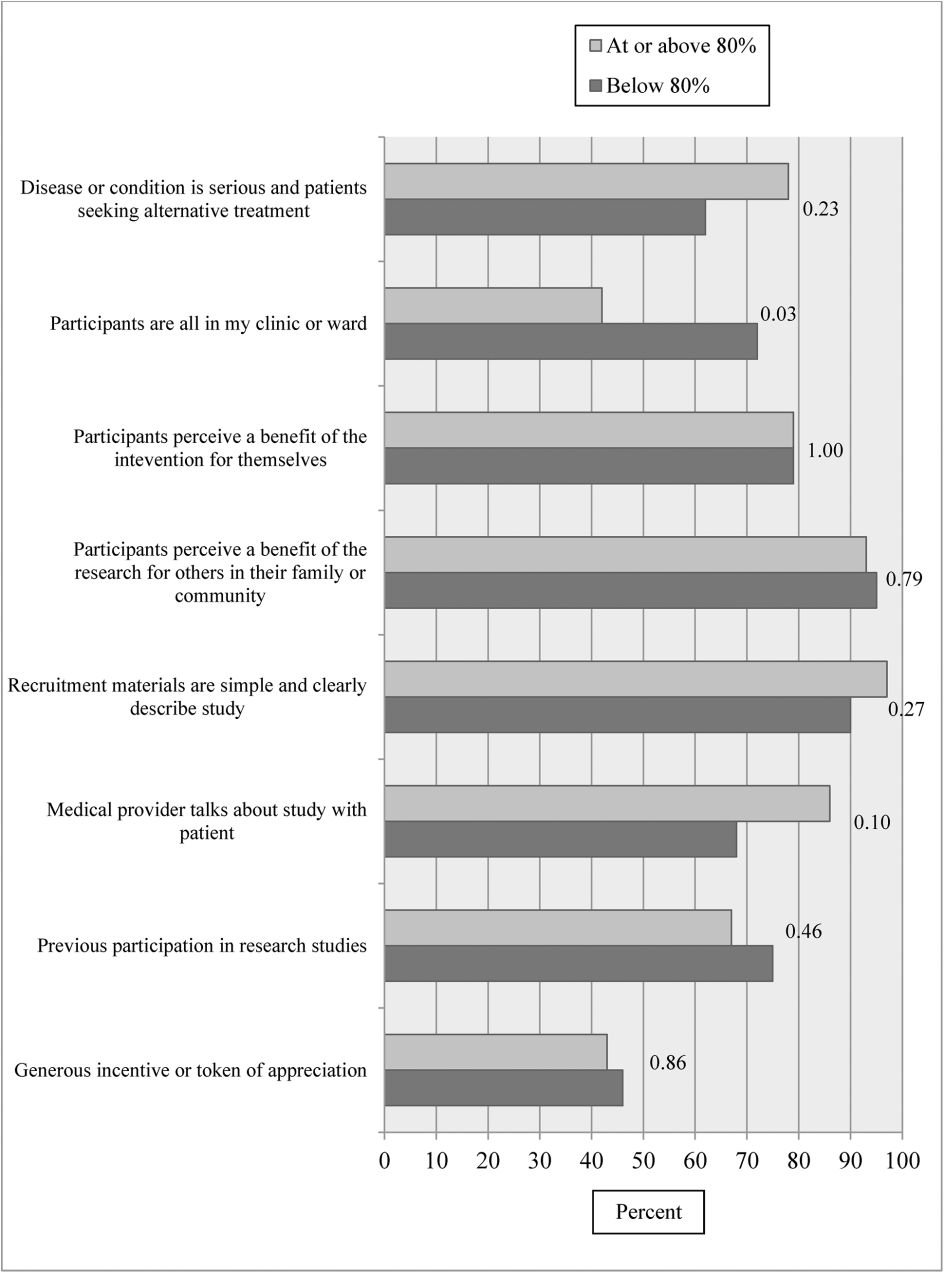
Percent of investigators agreeing with each facilitator according to reaching target enrollment among actively recruiting protocols.

Among those studies actively recruiting at the time of the survey, none of the perceived barriers differed significantly between those who reached vs. did not reach their 80% target (Fig 5). The only significant difference was in the perceived facilitator of “all participants being in their outpatient clinic or ward” (Fig 6), which was less often felt to be a facilitator by investigators who were reaching compared to not reaching 80% of their target (42% vs. 72%, respectively; p = 0.03).

## Discussion

Our survey found that a substantial proportion (34%) of protocols did not achieve 80% or more of their target enrollment at the time enrollment was closed, and an even greater proportion (65%) of actively recruiting studies were not meeting their enrollment targets during implementation. Many studies experienced substantial delays in recruiting with half of studies experiencing a delay of 6 or more months in their recruitment timeline. Among those factors measured, recruitment method appeared to be the most important and consistent modifiable factor affecting recruitment for studies closed to enrollment. The majority of investigators’ perceptions about potential barriers and facilitators did not differentiate studies with successful versus unsuccessful recruitment.

Several studies have reported low recruitment rates and study discontinuation due to low enrollment [11,12]. Among 1017 randomized clinical trial protocols from 6 research ethics boards in Switzerland, Germany and Canada, 253 (24.9%) were discontinued and half reached 40.9% or less of the target enrollment (Median 40.9; IQR 28.5 to 59.8). Beyond the potential adverse consequences on study validity, the costs of studies with low enrollment can be substantial. In a single academic medical center in the US, 31% of 837 terminated clinical studies were due to low enrollment [13]. The total administrative institutional costs for the 210 low enrolling studies that also underwent full board review was nearly $1 million dollars during a one-year period, and the costs for start- up were twice as high as those for maintenance ($637,080 vs. $349, 875) [13]. Given the substantial costs to initiate and implement clinical research, feasibility studies as well as frequent monitoring of enrollment are essential to enhance study quality as well as prevent the use of often limited institutional resources [13].

We found, not unexpectedly, that the recruitment method is associated with higher study enrollment rates; recruitment in person may allow for a more participant centered approach. Recruiting in person can provide a more personalized introduction to the study and participant experience with an opportunity for potential participants to obtain a clearer explanation of the study and have questions fully addressed. In a survey of 4961 adult research participants from 15 U.S. based major academic medical centers funded by the National Institute of Health, research participants rated their overall experience very highly when they felt investigators and nurses treated them with respect, listened to them, and gave them understandable answers to their questions [14]. Along these lines, two key in-person recruitment strategies, recruiter flexibility and building rapport with patients, were found to increase recruitment of patients in a study of colorectal cancer screening conducted in 25 adult primary care practices [15]. Furthermore, in- person recruitment may provide an opportunity to promote the integration of the research and clinical teams and make for a more seamless participant experience. Higher consent rates for research have been observed in a pediatric Intensive Care Unit setting when a research assistant was introduced by a member of the clinical team prior to approaching the family (89.7% vs. 77.7% respectively; p = 0.04) [16]. Saldana et al [17] also found a higher recruitment rate in pediatric pharmacogenetic studies when there was an ongoing study team-patient relationship (90.7% vs. 46.5%, respectively) and when there was active involvement of the research team in clinical care (81.8% vs. 43.7%, respectively). In our study, we did not assess the roles of staff who participated in recruiting, or how the participants were approached; however, these may be additional factors to consider when designing successful recruitment strategies in clinical research.

In our study, several other potentially modifiable factors that related to study complexity or participant burden, including the number of study visits and study duration as well as investigator experience were suggestive of being meaningfully related to recruitment. Investigator experience may be associated with more practical study designs and more effective problem solving early on during the study implementation phase. In this manner, government funded studies, which are more often investigator initiated, are more likely to have low enrollment than industry funded studies (53.6% vs. 38% p<0.001) [13]. This may be because industry sponsored trials closely assess investigator qualifications to ensure they are qualified and adequately experienced [12]. This reinforces the importance of having senior investigators closely oversee and mentor junior investigators on the science as well as operational aspects of a study. Clarifying these associations in larger samples will be important, as a great deal of investigator-initiated studies drive scientific discoveries and biomedical research in academic medical centers.

The positive association between successful recruitment and lack of funding is unexpected, but this may be because unfunded studies are less complex and thus have a lower participant burden. In our sample, unfunded studies were much more likely to be observational when compared to funded studies suggesting they were less complex. Kitterman et al [13] similarly found that expedited or exempt studies were less likely to be low enrolling than full board review studies, the latter which were more often therapeutic and more complex (19.2% vs. 45.2%, respectively; p = 0.001). Higher refusal rates were also observed when physicians enrolling participants in 6 pediatric clinical investigation centers in France perceived the study as generating heavy practical burden on the subject or family (OR 1.3; 95% CI 1.17–1.45) [18]. Several studies have identified time constraints and study complexity including difficulty finding eligible patients as major barriers to recruitment [4,5,9,10]. Perceived restrictive eligibility criteria and long study duration, both related to complexity and participant burden, appeared to distinguish successful recruitment in our data. Focusing the objectives and streamlining the interventions and measurements in studies to minimize study complexity, when possible, may be an important consideration for successful recruitment.

Our study has some limitations. The data is self-reported and response rates are moderate with the potential for non-response bias. The gift card was likely not a significant incentive to participate. We are unable to characterize the type and magnitude of non-response bias that may be present in our sample due to lack of data on non-respondents. Given the small number of protocols that were trials in our sample, it is possible that non-respondents were more likely to be those with clinical trial protocols preventing us from reliably evaluating factors associated with successful recruitment in this subgroup. The use of a single hospital also limits generalizability, and the relatively small sample size provided limited statistical power and precision. However, the lack of observing significant relationships with other factors measured may also be due to the heterogeneous nature of the studies in our sample. Recruitment success for some variables may be highly specific to the study purpose and population.

## Conclusion

We have shown that recruiting in person may promote reaching an acceptable target enrollment in pediatric clinical research. This may not be unique to pediatrics but rather a cross cutting theme and approach to facilitate recruitment and participation in adult and pediatric research. Given that the success of research depends on having an adequate number of participants and nearly one third of the staff’s work time spent running a clinical trial is devoted to recruiting participants [6], its importance cannot be understated. Strategies to promote successful recruitment should be considered at the outset, when designing, staffing and budgeting for a study. Future research is needed on larger and more diverse samples to gain a better understanding of how the characteristics and qualifications of the individuals who conduct recruitment influence participant enrollment and how best to approach patients and families for their participation in clinical research studies.

## Supporting Information

S1 File Regression Results. (DOCX)

S2 File Survey. (DOC)

## Abbreviations

IRB: Institutional Review Board
CCI: Committee on Clinical Investigation
BCH: Boston Children’s Hospital
