# Supplementary material for: Factors Associated with Clinical Research Recruitment in a Pediatric Academic Medical Center—A Web-Based Survey
Source: PLoS One. 2015 Oct 16;10(10):e0140768. doi: 10.1371/journal.pone.0140768 (PMC4608599; doi:10.1371/journal.pone.0140768)
Supplement: S1 File — (DOCX) [file pone.0140768.s001.docx]

**Regression Results**

**Table 1: Results of logistic regression multivariable model to examine factors associated with achieving 80% or more**

**of target enrollment among closed to enrollment protocols**

| **Variable** | **Beta** | **S.E. Beta** | **Wald** | **P-value** | **Odds Ratio**  **Exp(Beta)** | **95%CI** |
| --- | --- | --- | --- | --- | --- | --- |
| Study Design  (observational vs. clinical trial) | -0.448 | 0.575 | 0.606 | 0.436 | 0.639 | 0.207 , 1.972 |
| Recruitment Method  (In person vs. Other Methods) | 1.514 | 0.640 | 5.599 | 0.018 | 4.546 | 1.297, 15.933 |
| Funded  (Yes vs. No) | -1.299 | 0.702 | 3.424 | 0.064 | 0.273 | 0.069, 1.080 |
| More than one study visit  (Yes vs. No ) | -0.596 | 0.559 | 1.137 | 0.286 | 0.551 | 0.184, 1.647 |
| Constant | 0.926 | 0.666 | 1.934 | 0.164 | 2.524 | - |

Multivariable model includes study design, recruitment method and study visit; constant is also included in the model

**Table 2: Results of logistic regression multivariable model to examine factors associated with achieving 50% or more**

**of target enrollment among closed to enrollment protocols**

| **Variable** | **Beta** | **S.E. Beta** | **Wald** | **P-value** | **Odds Ratio**  **Exp(Beta)** | **95%CI** |
| --- | --- | --- | --- | --- | --- | --- |
| Study Design  (observational vs. clinical trial) | -1.022 | 0.700 | 2.129 | 0.145 | 0.360 | 0.091, 1.420 |
| Recruitment Method  (In person vs. Other Methods) | 2.205 | 0.749 | 8.673 | 0.003 | 9.070 | 2.091 , 39.352 |
| Years Clinical Research Experience  (5-9 years vs. 0-4 years) | -0.856 | 0.822 | 1.083 | 0.298 | 0.425 | 0.085, 2.130 |
| Years Clinical Research Experience  (10 or more years vs. 0-4 years) | 0.975 | 0.948 | 1.057 | 0.304 | 2.650 | 0.413, 16.990 |
| Constant | 0.445 | 0.846 | 0.277 | 0.599 | 1.561 |  |

Multivariable model includes study design, recruitment method and years of clinical research experience; constant is also

included in the model
