# Supplementary material for: Factors Associated with Clinical Research Recruitment in a Pediatric Academic Medical Center—A Web-Based Survey
Source: PLoS One. 2015 Oct 16;10(10):e0140768. doi: 10.1371/journal.pone.0140768 (PMC4608599; doi:10.1371/journal.pone.0140768)
Supplement: S2 File — (DOC) [file pone.0140768.s002.doc]

**[SURVEY]**

Welcome to the Survey on Recruitment of Research Participants!

While you might participate in a number of clinical research studies, only one study was chosen at random. Please consider **only** {study name} when answering this survey.

Thank you so much, in advance, for your participation in this survey!

Please click “next” to continue.

[Next]

Would you be willing to give permission to review your study’s consent form to help us validate some of the information you provide on this survey? Please check one response:

□ Yes, you may review my consent form on the Informed Consent Library

□ No, I would prefer not to provide access to my Informed Consent

What is the recruitment status of this study?

□ Not yet recruiting 🡪 END

□ Completed or currently recruiting 🡪 1

1. What type of study is [STUDY TITLE]?
   1. Drug trial
   2. Device trial
   3. Behavioral intervention
   4. Research registry
   5. Cross-sectional Survey
   6. Case-control
   7. Prospective Cohort
   8. Other (please specify: _______________________________________)

2. Is this a randomized trial?

□ Yes

□ No

3. Is there a placebo or active control?

□Yes, placebo

□Yes, active control

□No, neither

□Other (please specify) _______

4a. What is the minimum age of subjects that you are studying?

[select age with drop down menu] □Years □Months □Days

4b. What is the maximum age of subjects that you are studying?

[select age with drop down menu] □Years □Months □ Days

5. Does this study include males, females, or both?

□Males only

□Females only

□Both males and females

1. Do you recruit subjects with a specific disease or condition?

□ Yes-🡪6a

□ No 🡪 7

6a. What disease/condition do the study subjects have?

6b. What is the risk of death and/or disability and/or significant medical complications of this disease/condition?

□ High

□Moderate

□Minimal

□None

6c. Is there an effective treatment for this disease/condition?

□Yes

□No

7. Is the patient population of this study symptomatic or asymptomatic?

□Symptomatic

□Asymptomatic

□Both symptomatic and asymptomatic

8. Do you recruit healthy volunteers for this study?

□Yes

□No

9. What is the IRB designated risk/benefit determination associated with participation in this protocol?

□ No more than Minimal Risk; Potential for Direct Benefit

□ No more than Minimal Risk; No Potential for Direct Benefit

□ Greater than Minimal Risk; Potential for Direct Benefit

□ Greater than Minimal Risk; No Potential for Direct Benefit, but likely to yield generalizable knowledge about the subject’s condition

10. How many study visits are research only?

□ All

□ Most

□ Some

□ None

11. Where do study visits occur? (check all that apply)

□ Children's Hospital Boston Main Campus Inpatient Units

□ Children's Hospital Boston Main Campus Ambulatory Clinics

□ Children's Hospital Boston Clinical and Translational Study Unit (CTSU)

□ Children’s Hospital Boston Waltham satellite

□ Other non-Children’s Hospital Boston clinics/medical sites

□ In their own home

□ Non-medical site (i.e. community, schools)

□ Web, mail, or telephone survey

□ None of these places

12. What is the duration of a typical study visit? [select answer from drop down menu]

□ hours___ ___ □ days

13. Is more than one study visit required?

□Yes 🡪 13a

□No 🡪 14

13a. How many study visits are typically required? [select answer from drop down menu]

13b. What is the duration of the study for participants? (Indicate the maximum duration for a participant if duration varies due to the specifics of the study protocol)

[select answer from drop down menu] □days □weeks □ years

14. Are participant incentives or tokens of appreciation provided?

□Yes 🡪14a

□No 🡪15

14a. What types of incentives or tokens of appreciation are provided? (Check all that apply)

□Gift card

□Gift

□Parking voucher

□Transportation reimbursement

□Cash

□Other (please specify) __________

14b. What is the approximate total dollar value of the incentives if all study visits are completed? (Not including transportation reimbursement or parking)

$ _________

15. Which of the following best describes where you stand in your recruitment process?

□ Currently recruiting 🡪 Section A

□ Completed recruiting 🡪 Section B

16. What is your target enrollment number for this study? ___ ___ ___

17. How many participants are currently enrolled in this study? ___ ___ ___

18. How many participants have dropped out of this study or have been lost to follow up? ____

19 How many participants have completed the study? _____________________

20. When did you begin recruitment? (mm/yyyy)? _________________

21. When do you plan to end recruitment? (mm/yyyy)? _____________

22. Have you experienced delays in your study recruitment timeline?

□Yes 🡪 22a

□ No 🡪 23

22a. (Section A) By how many months are you now delayed from your original timeline? [select from drop down menu]

23. (Section A) What types of recruitment methods do you use in this study? (Please select all that you have previously used and/or currently use in the study)

□ In person

□ Newspaper ads

□ Internet ads

□ Radio/TV ads

□ Flyers/brochures

□ Referrals

□ Telephone recruitment

□ Mail recruitment

□ Email recruitment

□ CHB Connect

□ Other (please specify) ____________________________________________________

24. (Section A) Is this study funded?

□ Yes 🡪24a

□ No 🡪 25

24a. (Section A) To what extent do you feel that this study is adequately funded to achieve your study goals?

□ Well funded

□ Moderately well funded

□ Not well funded

25. (Section A) What percent of the principal investigator’s time is dedicated to working on this study? ____%

26. (Section A) Do you have a study coordinator for this study?

□ Yes 🡪26a

□ No 🡪27

26a. (Section A) How many FTEs of study coordinator/assistant time support the study?

□ >0.0 to 0.5

□ >0.5 to 1.0

□ >1.0 to 2.0

□ >2.0 to 3.0

□ More than 3.0

26b, (Section A) At the start of the study, how many years of experience did your most senior coordinator have?

□ None

□ >0-5 years

□ 6-10 years

□ Greater than 10 years

27. (Section A) What is the ethnicity of the study population?

□ Hispanic or Latino

□ Not Hispanic or Latino

□ Both

28. (Section A) What is the race of the study population? (check all that apply)

□ White/Caucasian

□ Black/African America

□ Asian

□ Native Hawaiian or Pacific Islander

□ American Indian or Alaska Native

□ Other (please specify) ____________________________________________________

16. (Section B) What was your target enrollment number for this study? ______

17. (Section B) How many participants were enrolled when this study ended? _____

18. (Section B) How many participants completed this study? __________

19. (Section B) How many participants dropped out of this study or were lost to follow up? _______

1. (Section B) When did you begin recruitment (mm/yyyy)?

20a. (Section B) When did you end recruitment (mm/yyyy)?

1. (Section B) Did you experience delays in your study recruitment timeline?

□ Yes 🡪21a

□ No 🡪22

21a. (Section B) By how many months are you now delayed from your original timeline? [select from drop down menu]

22. (Section B) Did the study end early or stop prematurely?

□ Yes 🡪 22a

□ No 🡪 23

22a. (Section B) Why did the study end early or stop prematurely?

□ Interim analysis for early efficacy or futility

□ Data and Safety Monitoring Board stopped study for other reasons

□ Lack of funding

□ Other (please specify) ________

23. (Section B) What types of recruitment methods do you use in this study? (Check all that apply)

□ In person

□ Newspaper ads

□ Internet ads

□ Radio/TV ads

□ Flyers/brochures

□ Referrals

□ Telephone recruitment

□ Mail recruitment

□ Email recruitment

□ CHB Connect

□ Other (please specify) ____________________________________________________

24. (Section B) Was your study funded?

□ Yes 🡪24a

□ No 🡪 25

24a. (Section B) To what extent do you feel that this study was adequately funded to achieve your study goals?

□ Well funded

□ Moderately well funded

□ Not well funded

1. (Section B) What percent of the principal investigator’s time was dedicated to working on this study? ____%
2. (Section B) Did you have a study coordinator for this study?

□ Yes 🡪 26a

□ No 🡪 27

26a. (Section B) How many FTEs of study coordinator/assistant time support the study?

□ >0.0 to 0.5

□ >0.5 to 1.0

□ >1.0 to 2.0

□ >2.0 to 3.0

□ More than 3.0

26b. (Section B) At the start of the study, how many years of experience did your most senior coordinator have?

□ None

□ >0-5 years

□ 6-10 years

□ Greater than 10 years

1. What was the ethnicity of the study population?

□ Hispanic or Latino

□ Not Hispanic or Latino

□ Both

1. What was the race of the study population? (check all that apply)

□ White/Caucasian

□ Black/African America

□ Asian

□ Native Hawaiian or Pacific Islander

□ American Indian or Alaska Native

□ Other (please specify) ____________________________________________________

29. (Resume all participants) With regard to your study that was identified in the email invitation,, please indicate how strongly you agree or disagree with each of the following statements.

I believe participant recruitment is **difficult** in this study because…

|  | **Strongly agree** | **Agree** | **Disagree** | **Strongly disagree** | **Not applicable** |
| --- | --- | --- | --- | --- | --- |
| I do not have time for it myself. |  |  |  |  |  |
| The opportunity to identify eligible participants is frequently missed. |  |  |  |  |  |
| Patients/families are concerned about risks associated with the research intervention/treatment |  |  |  |  |  |
| Patients/families prefer the standard of care |  |  |  |  |  |

29a. I believe participant recruitment is **difficult** in this study because…

|  | **Strongly agree** | **Agree** | **Disagree** | **Strongly disagree** | **Not applicable** |
| --- | --- | --- | --- | --- | --- |
| Geographic or transportation constraints get in the way of participation |  |  |  |  |  |
| The duration of research visits involve a significant time commitment |  |  |  |  |  |
| The study duration is too long |  |  |  |  |  |
| The intervention is burdensome |  |  |  |  |  |

29b. I believe participant recruitment is **difficult** in this study because…

|  | **Strongly agree** | **Agree** | **Disagree** | **Strongly disagree** | **Not applicable** |
| --- | --- | --- | --- | --- | --- |
| Patients/families are concerned it will negatively influence the doctor-patient relationship |  |  |  |  |  |
| Providers are concerned it will negatively influence the doctor-patient relationship |  |  |  |  |  |
| The study population is not English speaking |  |  |  |  |  |
| Too restrictive eligibility/inclusion criteria |  |  |  |  |  |

29c. Please describe up to three factors that make participation recruitment difficult in this study.

______________________________________________________________________________________________________________________________________________________________________________________________________________________________________________________

30. I believe participant recruitment is **successful** in this study when …

|  | **Strongly agree** | **Agree** | **Disagree** | **Strongly disagree** | **Not applicable** |
| --- | --- | --- | --- | --- | --- |
| A generous incentive or token of appreciation is given |  |  |  |  |  |
| A participant has previous participation in research studies |  |  |  |  |  |
| I talk about the study with the patient, because I am his/her medical provider |  |  |  |  |  |
| Recruitment materials are simple and clearly describe the study. |  |  |  |  |  |

30a. I believe participant recruitment is **successful** in this study when …

|  | **Strongly agree** | **Agree** | **Disagree** | **Strongly disagree** | **Not applicable** |
| --- | --- | --- | --- | --- | --- |
| Participants perceive a benefit of the research for others in their family or community |  |  |  |  |  |
| Participants perceive a benefit of the intervention for themselves |  |  |  |  |  |
| Participants are all in my outpatient clinic or inpatient ward |  |  |  |  |  |
| The disease/condition of interest is serious and patients are seeking alternative treatment |  |  |  |  |  |

30b. Please describe up to three factors that make participant recruitment successful in this study:

31. What would help investigators meet participant recruitment goals at Children’s Hospital Boston?

________________________________________________________________________________________________________________________________________________________________________________________________________________________________________________________________________________________________________________________________________

The following questions will help us to understand who our participants are, please take a moment to fill out this information.

D1. With which Children's Hospital Boston department are you **primarily** affiliated?

□ Cardiology

□ Cardiac surgery

□ Dental

□ Medicine

□ Neurology

□ Neurosurgery

□ Ophthalmology

□ Orthopedics/Orthopedic surgery

□ Otolaryngology

□ Plastic surgery

□ Psychiatry

□ Surgery

□ Urology

□ Other (please specify): _____________________

□ Prefer not to answer

D2. What is your position at Children's Hospital Boston?

□ Physician (Attending)

□ Physician (Trainee)

□ Nurse Scientist

□ Physician Assistant

□ Social Worker

□ Psychologist

□ Dentist

□ Other (please specify): _____________________

□ Prefer not to answer

D3. What is your faculty rank at Children's Hospital Boston?

□ Instructor

□ Assistant Professor

□ Associate Professor

□ Professor

□ Do not have a faculty appointment

□ Prefer not to answer

D4. Have you had any of the following kinds of training in clinical research methods? (check all that apply)

□ Doctoral training in research methods

□ Master in Public Health

□ Other Master’s degree in research methods

□ Clinical research training seminars offered through CHB

□ Clinical research training seminars offered outside of CHB

□ Other (please specify): _____________________

□ No clinical research methods training or degree

□ Prefer not to answer

D5. How many years have you been a Principal Investigator? (If you have never been the Principal Investigator of a study, please enter “0”) ___________________________

D6. How many years have you been involved in clinical research that involves human subject recruitment? (If less than one year, enter “0”

D7. How many years have you worked at Children’s Hospital Boston? (If less than one year, enter “0”) __________________

D8. How many clinical research studies have you implemented to date?

D9. What is your gender?

□ Male

□ Female

□ Prefer not to answer

D10. What age group do you belong to?

□ 20-30 years

□ 31-40 years

□ 41-50 years

□ 51-60 years

□ 61-70 years

□ 71-80 years

□ 81+ years

□ Prefer not to answer

E1. What is the role of the person who completed this survey (select all that apply)?

□ Principal Investigator

□ Co-Investigator

□ Study Coordinator

□ Research Assistant

□ Project Director

□ Administrative Associate

□ Other (please specify) _____________

This is all of the questions we have for you to answer. Thank you for completing this survey; we

will send you a $20 Starbucks gift card as a token of our appreciation.

If you have any questions about this study, please feel free to contact Erica Denhoff at Erica.Denhoff@cardio.chboston.org.

Thank you for completing this survey. You may now close your browser window.
